# The combination of circle topology and leaky integrator neurons remarkably improves the performance of echo state network on time series prediction

**DOI:** 10.1371/journal.pone.0181816

**Published:** 2017-07-31

**Authors:** Fangzheng Xue, Qian Li, Xiumin Li

**Affiliations:** 1 Key Laboratory of Dependable Service Computing in Cyber Physical Society of Ministry of Education, Chongqing University, Chongqing 400044, China; 2 College of Automation, Chongqing University, Chongqing 400044, China; Tianjin University, CHINA

## Abstract

Recently, echo state network (ESN) has attracted a great deal of attention due to its high accuracy and efficient learning performance. Compared with the traditional random structure and classical sigmoid units, simple circle topology and leaky integrator neurons have more advantages on reservoir computing of ESN. In this paper, we propose a new model of ESN with both circle reservoir structure and leaky integrator units. By comparing the prediction capability on Mackey-Glass chaotic time series of four ESN models: classical ESN, circle ESN, traditional leaky integrator ESN, circle leaky integrator ESN, we find that our circle leaky integrator ESN shows significantly better performance than other ESNs with roughly 2 orders of magnitude reduction of the predictive error. Moreover, this model has stronger ability to approximate nonlinear dynamics and resist noise than conventional ESN and ESN with only simple circle structure or leaky integrator neurons. Our results show that the combination of circle topology and leaky integrator neurons can remarkably increase dynamical diversity and meanwhile decrease the correlation of reservoir states, which contribute to the significant improvement of computational performance of Echo state network on time series prediction.

## Introduction

Echo state network (ESN), one of the improved recurrent neural networks, has attracted extensive attention since proposed by Jaeger in 2002 [1]. Unlike recurrent neural network, ESN has a non-trainable sparse connected recurrent part (dynamic reservoir) as the hidden layer and only the output weight need to be trained. The internal weights and input weights of ESN are generated randomly and remain unchanged during training and testing. The readout training is a simple linear regression problem for supervised learning. Due to the simple method and high learning efficiency, ESN has been successfully applied to many fields, such as time series prediction tasks [2, 3], dynamic pattern classification [4–7], telephone traffic forecasting [8, 9], stock price prediction [10], speech recognition [11, 12], and so on.

Recently, many modified ESN models have been proposed from different aspects to enhance the network performance: (1) From the reservoir topology perspective, literature [13] successfully applied small-word network and scale-free network based on complex network theory to replace the random dynamic reservoir topology of ESN; a new scale-free and highly clustered ESN with both small-world feature and scale-free characteristic was proposed in [14, 15]; In [16] the authors adopted hierarchical reservoir to deal with multiscale input signal based on the error gradient descent method and decoupled reservoirs were applied to ESN in [17]. Contrary to a randomly initialized and fixed structure, [18, 19] used developmental self-organization approaches to regulate the synaptic and structural plasticity of the dynamic reservoir according to the specific tasks. In addition, various methods have been proposed for analysing time series by means of complex network [20, 21]. It has been shown that these approaches have advantage for characterizing real complex systems from nonlinear time series [22–24]. These studies provide insights for constructing reservoir topology of ESNs. (2) In the aspect of training algorithms, [25] applied ridge regression learning algorithm in ESN to solve the ill-condition matrix; In [26], the authors proposed a priori data-driven multi-cluster reservoir generation algorithm; A regularized variational Bayesian learning was learned in [27]. (3) In terms of reservoir neuron models, wavelet neurons were used in reservoir state update equation in [28] and filter neurons were adopted in [29]. (4) From the point of energy consumption, it has been found that the performance of network structure is not only related to the weight of network connectivity, but also to the energy utilization of the network behavior [30–33].

Specifically, in order to reduce the randomness of dynamical reservoir, a predefined singular value spectrum of the internal weight matrices is adopted in [34]. To further simplify the reservoir topology, literature [35] put forward several simple topologies: delay line reservoir (DLR), delay line reservoir with feedback connections (DLRB) and simple cycle reservoir (SCR). These three reservoir construction approaches are simple and deterministic to realize without the loss of performance compared to classical ESN for some learning tasks. To increase the diversities of the dynamic reservoir, [28] injected wavelet neurons into reservoir and assigned the sigmoid and wavelet neurons randomly. [36] replaced parts of sigmoid neurons with wavelet units in a circle structure at different injecting ratio and distribution interval. It has been proved that the hybrid circle reservoirs which contain two kinds of neurons have certain advantages over the simple circle structures with only one kind of neuron.

In [1] and [12], Jaeger also proposed that the internal neurons were not confined to sigmoid units and applied leaky integrator neurons to ESN. He pointed out some disadvantages presenting traditional sigmoid neurons: (1) The conventional sigmoid units did not have a time constant compared with the continuous leaky integrator neuron model; (2) The sigmoid units are memoryless since the next time state values of reservoir units in standard sigmoid networks do not depend on their previous values directly. Thus, it is more appropriate for us to apply the continuously and slowly changing systems using the continuous-time leaky integrator network.

In general, although the influence of either circle topology or leaky integrate neuron on reservoir computing have been studied in literature, the combined effect of these two factors have not been considered and carefully analyzed. Therefore, in this paper we apply both the circle structure and leaky integrator neuron to improve the computational performance of ESN, motivated by leaky integrator ESN introduced in [37] and the simple circle topology. Mackey-Glass time series is used to test the performance of four ESN networks: classical random ESN with sigmoid neurons, circle ESN with sigmoid neurons, random ESN with leaky integrator neurons, circle ESN with leaky integrator neurons. The prediction accuracy, nonlinear dynamics approximation ability and anti-noise capability are investigated respectively. The results show that our circle ESN with leaky integrator neurons remarkably outperform other ESNs. This work provides an efficient model of ESN with excellent performance and simple network structure, which is very meaningful for the broad application of ESN on various fields.

This paper is organized as follows. Section 2 describe four ESN models with different reservoir topology and neuron models. Section 3 briefly present experiment design including learning task, specific parameters setting and training process. Experiment results are shown in section 4. Finally, discussion and conclusion are made in Section 5.

## ESNS

### Traditional echo state network

**Fig 1:**
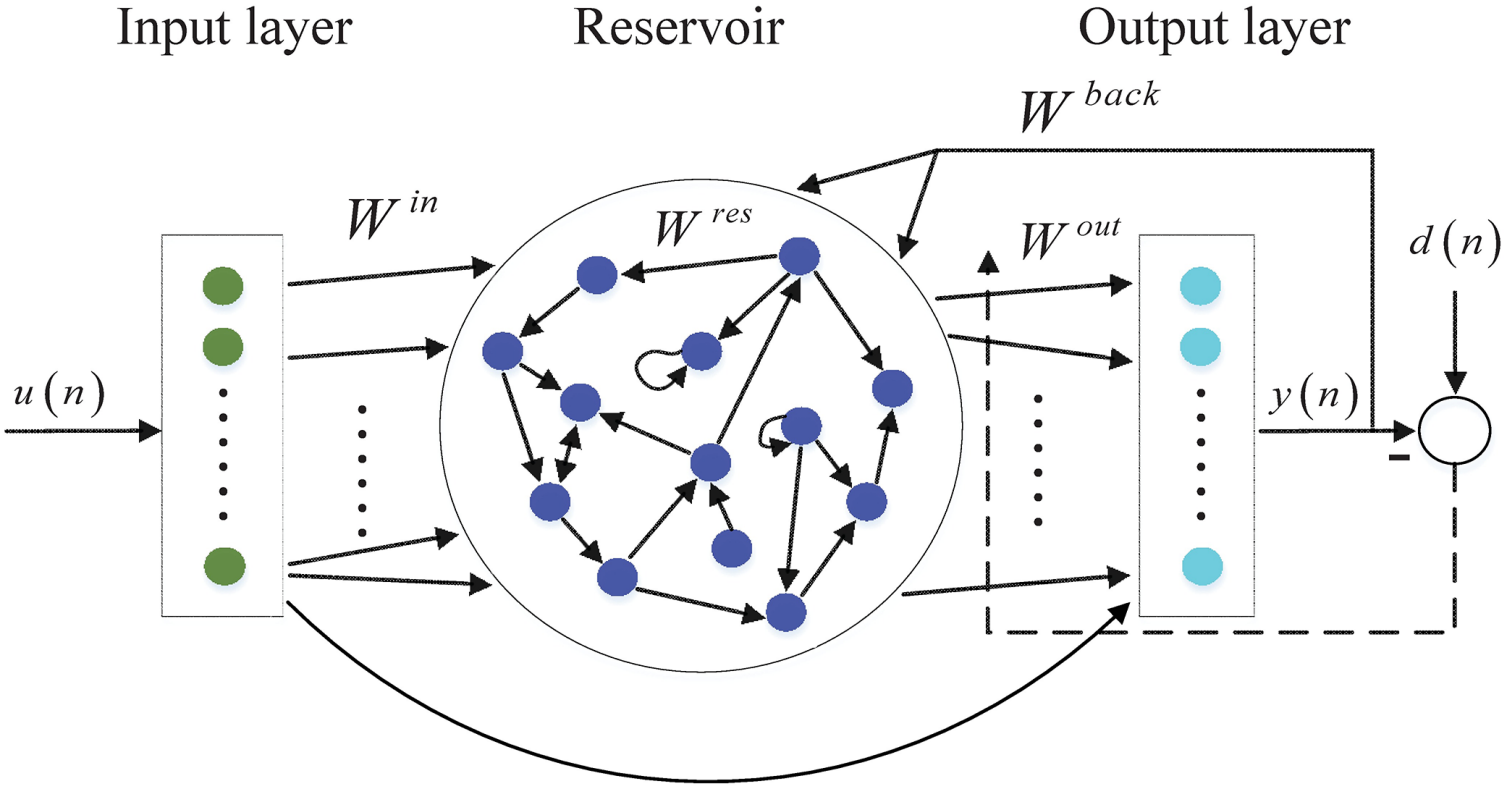
The regular echo state network model with random reservoir topology.

The architecture of ESN comprises an input layer, dynamical reservoir, and readout neuron. The traditional ESN has a randomly connected reservoir as illustrated in Fig 1. The ESNs are assumed to have K input neurons, N reservoir neurons, and L readout neurons, whose activation at time step *n* are denoted by **u**(*n*) = (*u*_1_(*n*), …*u*_*K*_(*n*))^*T*^, **x**(*n*) = (*x*_1_(*n*), …*x*_*N*_(*n*))^*T*^, and **y**(*n*) = (*y*_1_(*n*), …*y*_*L*_(*n*))^*T*^, respectively (In the rest of the paper, vectors are denoted by boldface lowercase letters, e.g., a, while matrices are denoted by boldface uppercase letters, e.g., A). The connection weights from the input neurons to reservoir neurons are given in a *N* × *K* matrix **W**^*in*^. The reservoir connection weights are collected in a *N* × *N* weight matrix **W**^*res*^. The connection weights from the input and reservoir neurons to the readout neurons are given in a *L* × (*K* + *N*) matrix **W**^*out*^. Furthermore, the connection weights projected back from the readout neurons to the reservoir neurons are given in a *N* × *L* matrix **W**^*back*^.

The reservoir is updated according to the following equation:

$$\begin{array}{c} x\left(n+1\right)=f\left(W^{in}u\left(n+1\right)+W^{res}x\left(n\right)+W^{back}y\left(n\right)+v\left(n\right)\right), \end{array}$$ (1)

where *f* is the activation function of the reservoir units (usually sigmoid function) and **v**(*n*) is a noise, we use tanh function as the internal neurons function here. The output is computed as:

$$\begin{array}{c} y\left(n+1\right)=f^{out}\left(W^{out}\left[u(n+1)|x(n+1)\right]\right), \end{array}$$ (2)

where *f*^*out*^ denotes the activation function of the output neuron, [**u**(*n* + 1)|**x**(*n* + 1)] denotes the concatenation vectors of the input and internal activation vectors and **W**^*out*^ is the output weight matrix that has been trained. In our paper, we use tanh function as the readout function as well.

In order to guarantee the echo state property (ESP), the spectral radius of the reservoir weight matrix must be kept below 1. This can be achieved by scaling the initialized sparse weight **W**_0_ into a new matrix **W**^*res*^ = *k* **W**_0_/|*σ*_max_|, where |*σ*_max_| denotes the spectral radius of **W**_0_ and the value of the scaling parameter *k* belong to (0, 1).

### ESNs with leaky integrator neurons

The traditional sigmoid units in reservoir have no working memory while the leaky integrator neurons have. Therefore, it is more proper to choose the leaky integrator units for learning the slowly and continuously changing dynamical systems. ESNs with leaky integrator neurons which is called leaky integrator ESN (LI-ESN) has been reported in [38]. The dynamic equation of LI-ESN is similar to the model proposed by Jaeger [37] described as follows:

$$\begin{array}{c} \dot{x}=\frac{1}{\zeta }\left(-ax+f(W^{in}u+W^{res}x+W^{back}y+v)\right), \end{array}$$ (3)

where *ζ* > 0 is the time constant. The positive constant a is the leaky decay rate and f denotes the activation function (we use sigmoid function as well). The matrix **W**^*in*^ represents the input weight matrix, **W**^*res*^ denotes the internal weight matrix, **W**^*back*^ is the feedback connection weight matrix. **u**, **x**, **y**, **v** denote the input vector, the reservoir state vector, the output vector and the noise vector, respectively. According to [39], the differential equation can be approximately turned into a difference equation as follows:

$$\begin{array}{c} x\left(n+1\right)=\left(1-a\right)x\left(n\right)+f\left(W^{in}u\left(n+1\right)+W^{res}x\left(n\right)+W^{back}y\left(n\right)+v\left(n\right)\right), \end{array}$$ (4)

where **x**(*n*) denotes the internal state vector at the sample time step *n*. The training method is similar to the classical ESN. The method for choosing the adequate parameter *a* will be discussed later.

### ESNs with Low complexity circle reservoir topology

**Fig 2:**
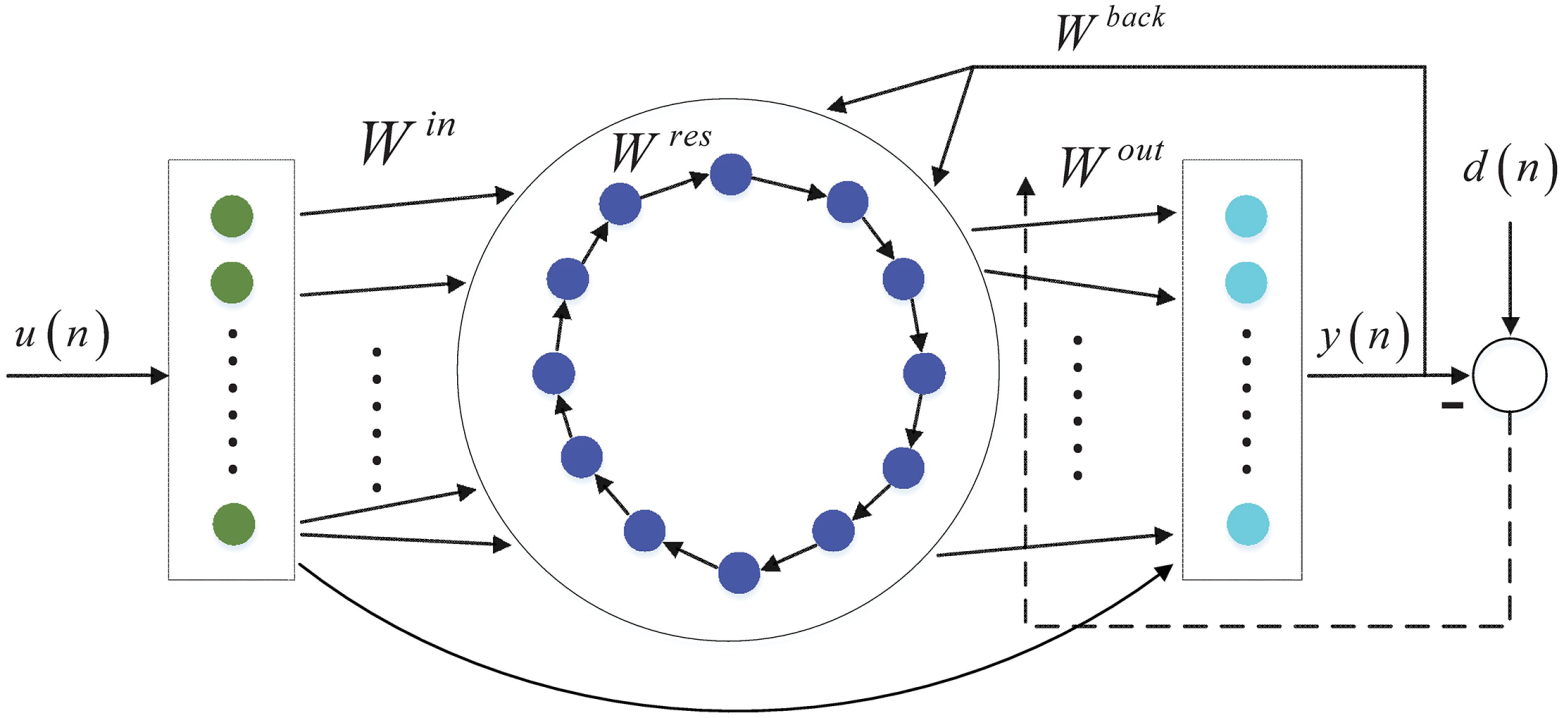
Network structure of circle reservoir topology with units to be sigmoid or leaky integrator neurons.

In this section, we introduce two ESNs with the simple circle reservoir topology shown in Fig 2 with sigmoid or leaky integrator neurons, which are called circle ESN (C-ESN) and circle LI-ESN (C-LI-ESN) respectively. Unlike the conventional ESN and LI-ESN, the input weight matrix **W**^*in*^ and the reservoir weight matrix **W**^*res*^ of C-ESN, C-LI-ESN have fixed weight values of *v* and *r*. The matrix of cycle reservoir and input weight matrix are described in Eqs (5) and (6), respectively. The values of *v*, *r* and *a* are adjusted depending on specific tasks. However, the reservoir update equation and the training process are done in similar ways as classical ESN and LI-ESN.

$$\begin{array}{c} W^{res}={\left[\begin{array}{cccccc} 0 & 0 & 0 & \cdots & 0 & r\\ r & 0 & 0 & \cdots & 0 & 0\\ \vdots & \vdots & \vdots & \ddots & \vdots & \vdots \\ 0 & 0 & 0 & \cdots & r & 0 \end{array}\right]}_{N\times N} \end{array}$$ (5)

$$\begin{array}{c} W^{in}={\left[\begin{array}{cccccc} v & v & v & \cdots & v & r\\ v & v & v & \cdots & v & v\\ \vdots & \vdots & \vdots & \ddots & \vdots & \vdots \\ v & v & v & \cdots & v & v \end{array}\right]}_{N\times K} \end{array}$$ (6)

In order to ensure the echo state property of the new C-LI-ESN, sufficient conditions are given as: (1) If |1 − (*a* − *σ*_max_)| < 1 (where *σ*_max_ is the maximal singular value of the **W**^*res*^), the C-LI-ESN has echo states; (2) If the spectral radius of the matrix $\tilde{W}=W^{res}+\left(1-a\right)E$ (where **E** is the identity matrix) exceed 1, the C-LI-ESN has no echo state property.

## Experiment design

### Learning task

**Fig 3:**
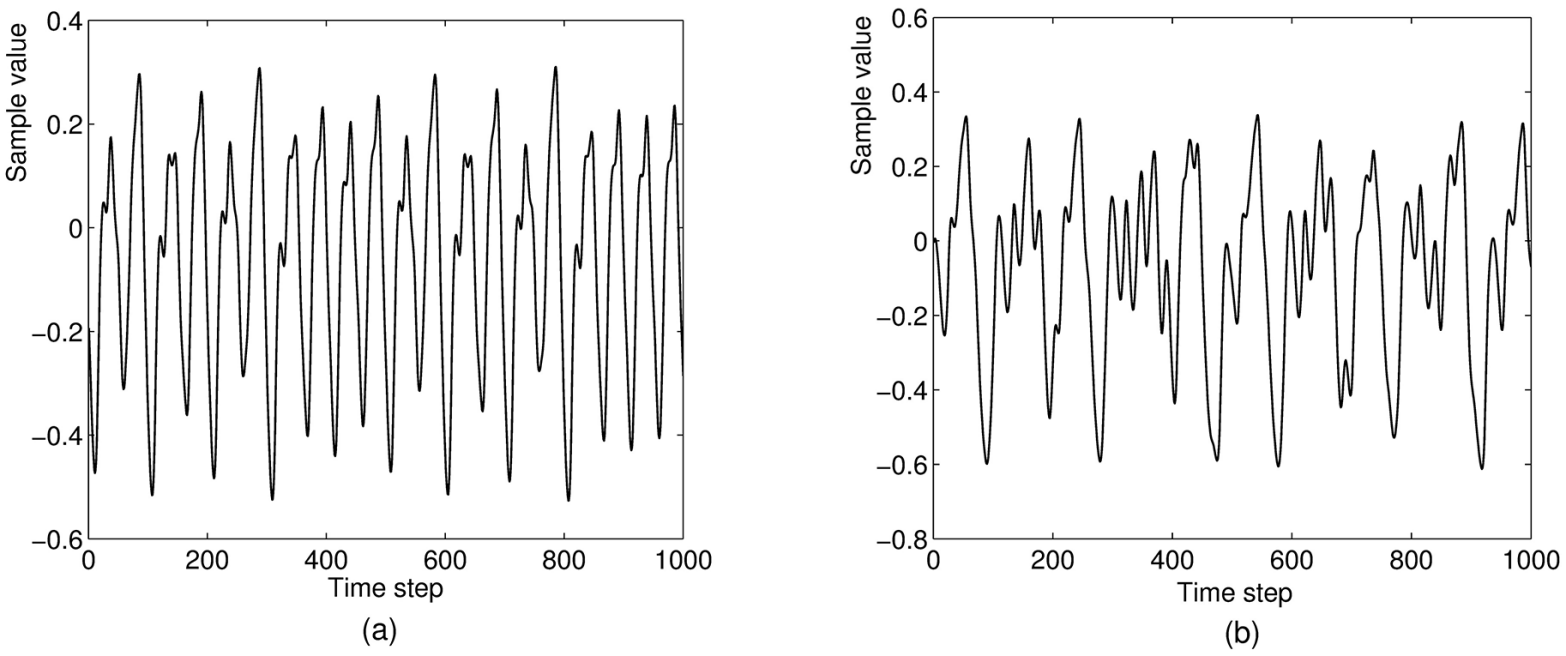
The Macky-Glass time series with (a) *τ* = 17 and (b) *τ* = 30.

In order to compare the performance of the four networks described above, we choose the widely used learning task: the Mackey-Glass system (MGS) time series prediction [5]. The discrete-time sequence equation of the MGS is defined as:

$$\begin{array}{c} y\left(t+1\right)=y\left(t\right)+\delta \left(\frac{0.2y(t-\frac{\tau }{\delta })}{1+y{\left(t-\frac{\tau }{\delta }\right)}^{10}}-0.1y\left(t\right)\right), \end{array}$$ (7)

where *δ* is the step size parameter which will always be set 0.1 with subsequently sub-sampling by 10, *τ* denotes the time delay parameter which determines the nonlinearity degree of the MGS. The MGS is a chaotic system if *τ* > 16.8. In this paper, two time series—one mild chaotic system and another wild system with *τ* = 17 and *τ* = 30 respectively will be used for prediction tasks. Fig 3 shows 1000-step subsequences of the two training sequences for these two cases.

### Network preparation

**Fig 4:**
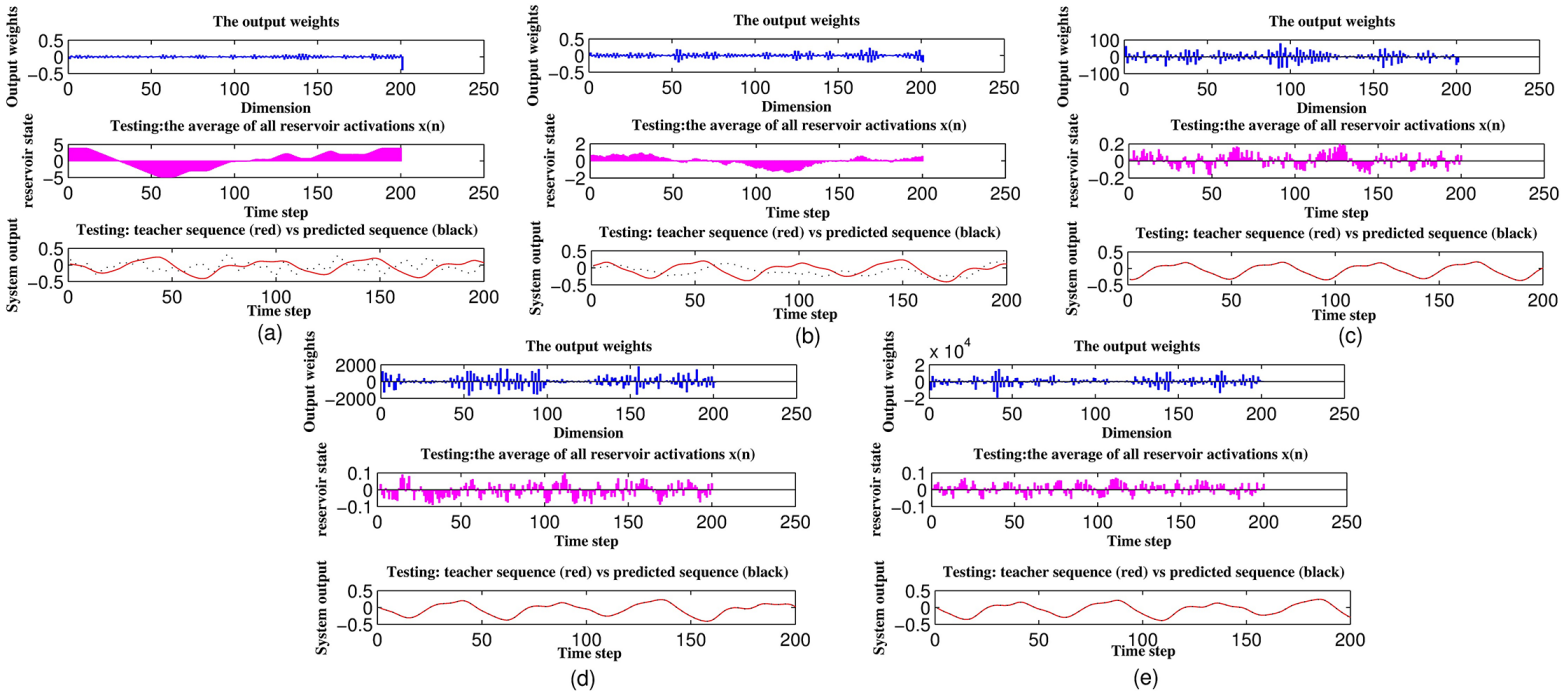
The influence of decay constant a on computing performance during the 200 steps of testing procedure. Top: W^*out*^ distribution; Middle: The average value of reservoir states; Bottom: Network output vs the teacher signal. (a) a = 0.2, *NRMSE*_84_ = inf; (b) a = 0.4, *NRMSE*_84_ = inf; (c) a = 0.6, *NRMSE*_84_ = 0.001; (d) a = 0.8, *NRMSE*_84_ = 0.0035; (e) a = 1.0, *NRMSE*_84_ = 0.0235.

In this paper, all of the four types of ESNs comprise one input unit, 200 reservoir units, and one readout neuron. The input is a fixed signal *u*(*n*) = 0.02. The magnitude of the noise is set 1e-10. For the traditional ESN, weight matrices **W**^*in*^ and **W**^*back*^ are drawn from a uniform distribution over [−1, 1], the spectral radius is set 0.85. For the circle topology structure and the LI-ESN, the optimal values for the fixed weight values *v*, *r* of **W**^*in*^, **W**^*res*^ and the decay constant *a* are considered as follows: 1) For LI-ESN, the matrix **W**^*res*^ is firstly re-scaled to 0.5 so that *σ*_max_ = 0.5; *a* is set 0.6 to ensure |1 − (*a* − *σ*_max_)| < 1 and the effective spectral radius of $\tilde{W}$ approximates 0.85; the connection values of **W**^*in*^ and **W**^*back*^ are the same as the traditional ESN. 2) For C-ESN, the input connection parameter *v* is chosen as 0.1 and the reservoir weight is 0.5 according to [36]. 3) For C-LI-ESN, we set the input weight *v* = 0.1 and reservoir weight *r* = 0.5, where the maximum singular value of **W**^*res*^ equals 0.5. In order to guarantee the echo state property, the decay constant *a* must be bigger than 0.5. In order to choose the optimal values of *a*, we compute the **W**^*out*^ distribution, average reservoir states and the network output respectively as shown in Fig 4. It shows that when *a* is less than 0.5, the predictive error can not converge to minor values near zero; However, when *a* approximates to 1, the internal states become smaller leading to very large values of output weights, which will reduce the performance of network prediction; Hence, the parameter *a* is set as 0.6 for C-LI-ESN. For C-ESN and C-LI-ESN, the weight matrices **W**^*back*^ are also drawn from a uniform distribution over [−1, 1].

### Training process

During this training process, only the output weight matrix **W**^*out*^ is trained by means of pseudo-inverse method based on collected reservoir states. Our networks learn a 3000-step training sequence d(n) (teacher sequence) and discard previous 1000 steps. That is to say, the total states are collected into a state matrix **M** from the last 2000 steps (from *d*(1001) to *d*(3000)). At the same time, the transfer-inverted version tanh^−1^(*d*(*n*)) is saved into a vector **T**. **M** and **T** are denoted as follows:

$$\begin{array}{c} M={\left[\begin{array}{cccccc} x_{1}\left(1001\right) & x_{2}\left(1001\right) & x_{3}\left(1001\right) & \cdots & x_{200}\left(1001\right) & u(1001)\\ x_{1}\left(1002\right) & x_{2}\left(1002\right) & x_{3}\left(1002\right) & \cdots & x_{200}\left(1002\right) & u(1002)\\ \vdots & \vdots & \vdots & \ddots & \vdots & \vdots \\ x_{1}\left(3000\right) & x_{2}\left(3000\right) & x_{3}\left(3000\right) & \cdots & x_{200}\left(3000\right) & u(3000) \end{array}\right]}_{2000\times 201} \end{array}$$ (8)

$$\begin{array}{c} T={\left[tanh^{-1}\left(d(1001)\right),tanh^{-1}\left(d(1002)\right)\cdots tanh^{-1}\left(d(3000)\right)\right]}^{T} \end{array}$$ (9)

where **x** is reservoir state and **u** is input signal. After time *n* = 3000, **W**^*out*^ is computed according to pseudo-inverse method:

$$\begin{array}{c} W^{out}={\left(M^{+}T\right)}^{T}. \end{array}$$ (10)

Once the output weights **W**^*out*^ are obtained, ESN is ready for testing its performance on time series prediction.

### Two testing criteria

Two testing criteria are employed as the performance measurements in this simulation: testMSE and *NRMSE*_84_. Firstly, ESNs run freely for another 200 steps from the last state of the training period. The performance of the four different types of ESNs are estimated by comparing the output with the desired teaching signal. The testing mean square error is calculated as follows:

$$\begin{array}{c} testMSE=\frac{1}{200}\sum_{n=3001}^{3200}{\left(d(n)-y(n)\right)}^{2} \end{array}$$ (11)

Secondly, the trained network run with a newly generated input sequence from the MGS system. The prediction performance is measured using the normalized RMSE at the 84th time step ((*NRMSE*_84_)). The internal state of the reservoir is initialized into 0 then updated under the newly generated input signal for 1000 steps. we run our network 50 times independently. The *NRMSE*_84_ is computed as follows:

$$\begin{array}{c} NRMSE_{84}=\sqrt{\frac{\sum_{i=1}^{50}{\left(y_{i}\left(n+84\right)-d_{i}\left(n+84\right)\right)}^{2}}{50\sigma^{2}}}, \end{array}$$ (12)

where *y*_*i*_[*n*] is the network output during the testing phase; *d*_*i*_[*n*] is the desired output during the testing phase; and *σ*^2^ is the variance of the desired output.

## Experiment results

According to the parameter settings for each network introduced in section 3.2, four different types of ESNs—traditional ESN, C-ESN, LI-ESN and C-LI-ESN are investigated in this section from the following three aspects: (1) prediction accuracy; (2) capability of nonlinear time series prediction; (3) anti-noise ability.

### Prediction accuracy

**Fig 5:**
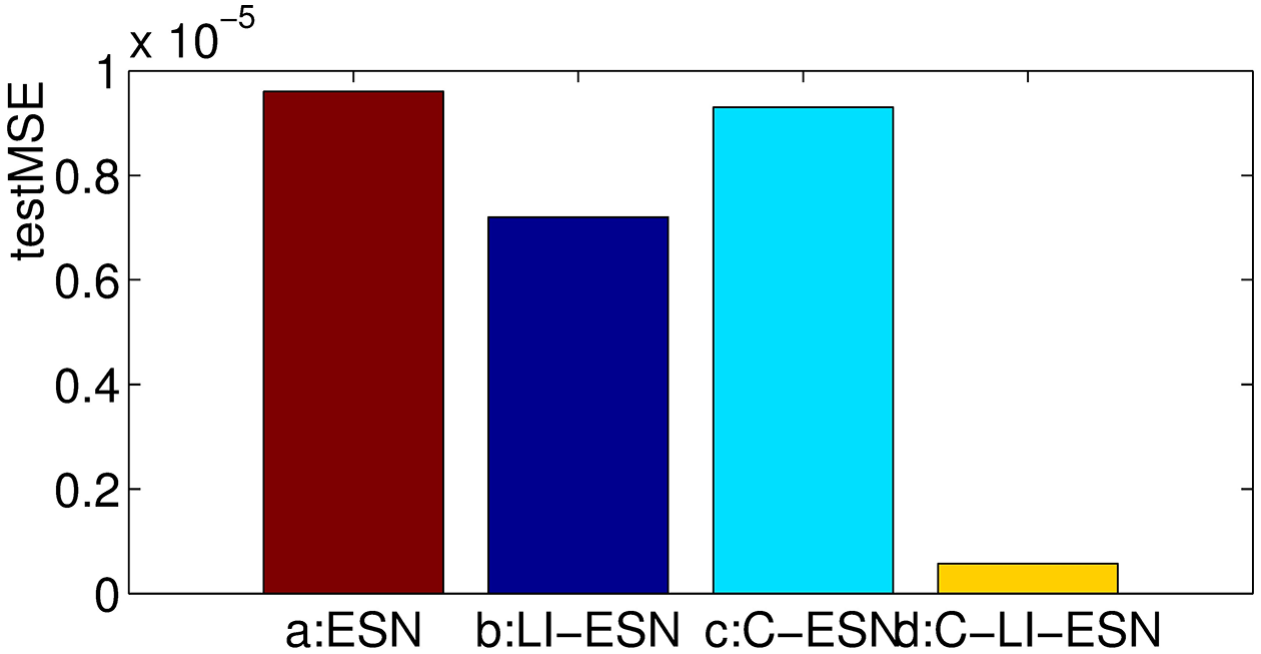
The *testMSEs* comparisons of prediction performance for four networks.

**Fig 6:**
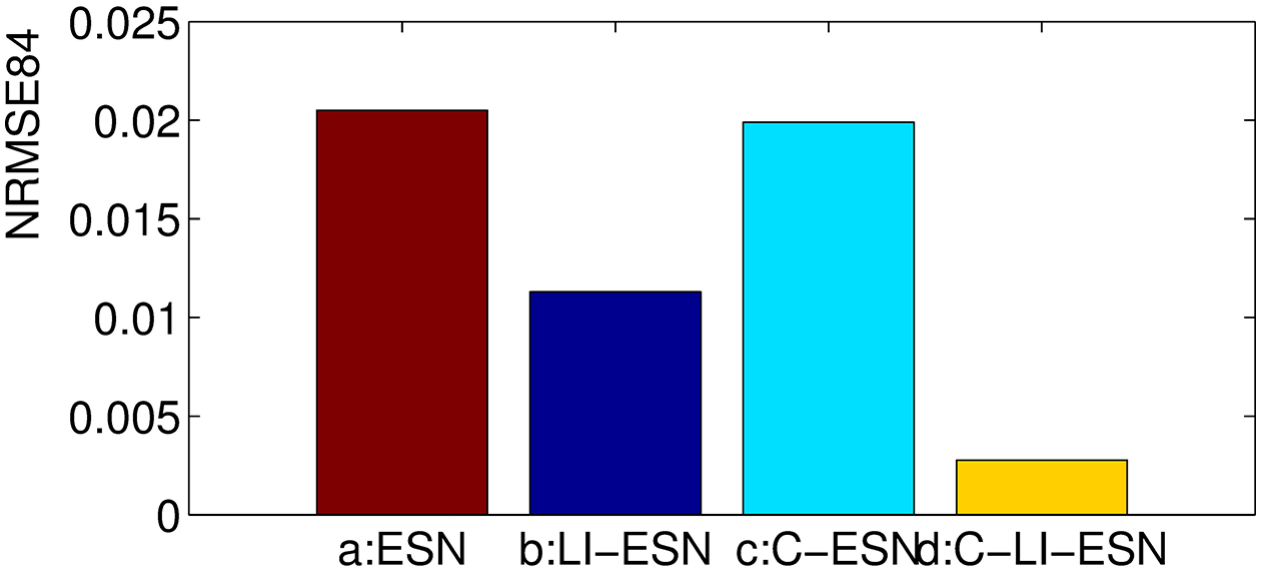
The *NRMSEs*_84_ comparisons of prediction performance for four networks.

In this simulation, the prediction task for MGS time series with *τ* = 17 is conducted by the four networks. We run the networks 20 times independently and average the results. The prediction performance of these four networks measured by *testMSE* and *NRMSE*_84_ are shown in Table 1, Figs 5 and 6. Obviously, it can be seen that the application of either cycle topology or leaky integrator neurons alone can make ESNs achieve better performance than traditional ESN with random reservoir and sigmoid neurons, which is consistent with the results reported in [15, 39]. Most importantly and surprisingly, the prediction accuracy of the C-LI-ESN greatly outperform other ESNs. The application of both cycle topology and leaky integrator neurons make the predictive error reduced by roughly 2 orders of magnitude. The combined interactions between the simple cycle topology and the leaky integrator units with memory of history reservoir state lead to richer dynamics and lower correlation of reservoir states, which may contribute to the remarkable enhancement of computational performance. We will discuss these in the following paragraphs in detail.

**Table 1:** The average results of 20 independent experiments for four networks.

Network models	test MSE	NRMSE84
ESN	9.6 × 10^−6^	2.05 × 10^−2^
LI-ESN	7.2 × 10^−6^	1.13 × 10^−2^
C-ESN	9.3 × 10^−6^	1.99 × 10^−2^
C-LI-ESN	2.4 × 10^−7^	1.84 × 10^−3^

**Fig 7:**
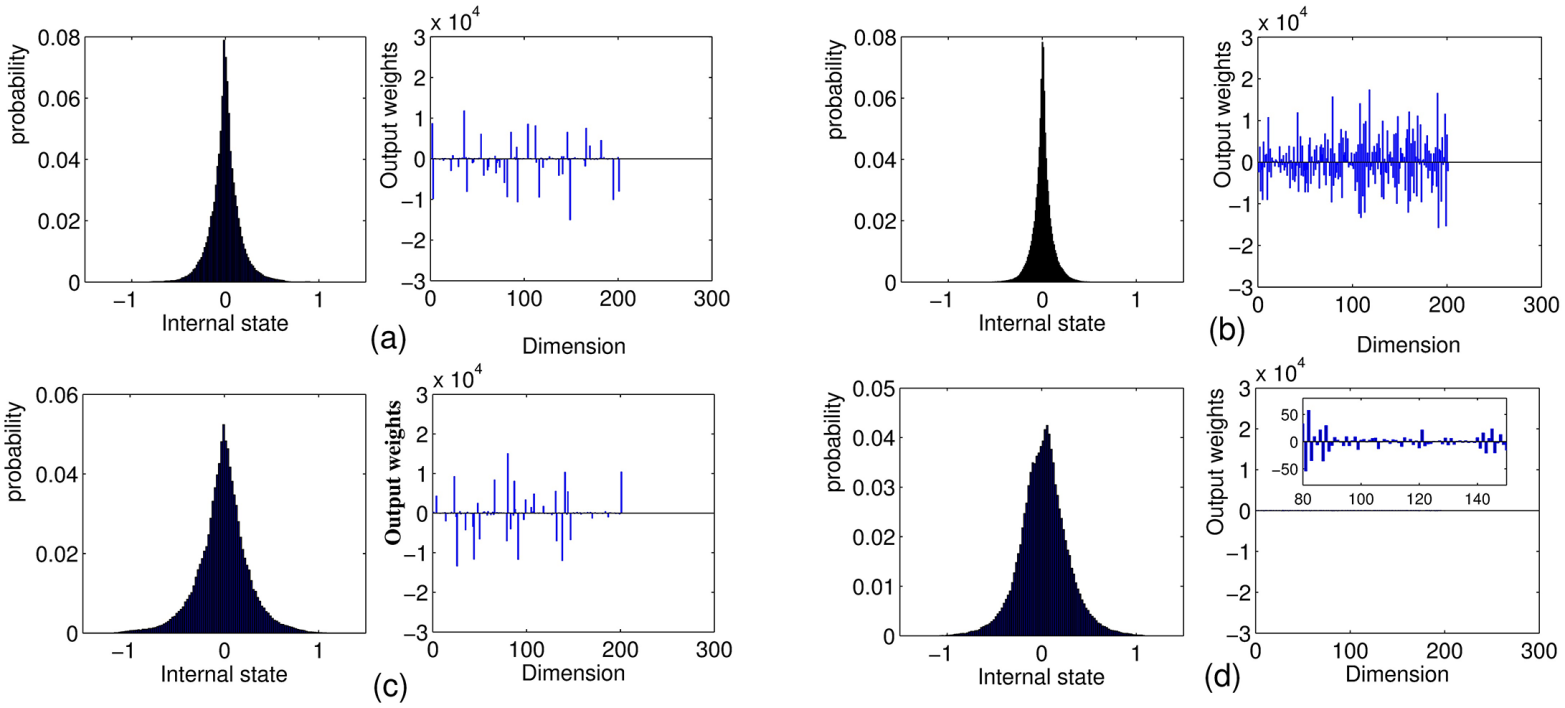
The probability distribution of all internal states and corresponding *W*^*out*^ distribution of four networks during training phase. (a) ESN (b) C-ESN (c) LI-ESN (d) C-LI-ESN.

In order to investigate the dynamical diversity of reservoir states, probability distribution of internal reservoir states and the corresponding **W**^*out*^ distribution of four networks are shown in Fig 7. It clearly shows that C-LI-ESN has much broader distribution of state values than other networks, indicating its richest dynamic characteristics of reservoir states. Besides, the trained **W**^*out*^ distribution of the C-LI-ESN is much smaller and in a more reasonable range compared with other three networks whose output weights are in the order of 100 or even larger. Based on [40], output weights should not be too large and the reasonable absolute values are not greater than 50.

**Fig 8:**
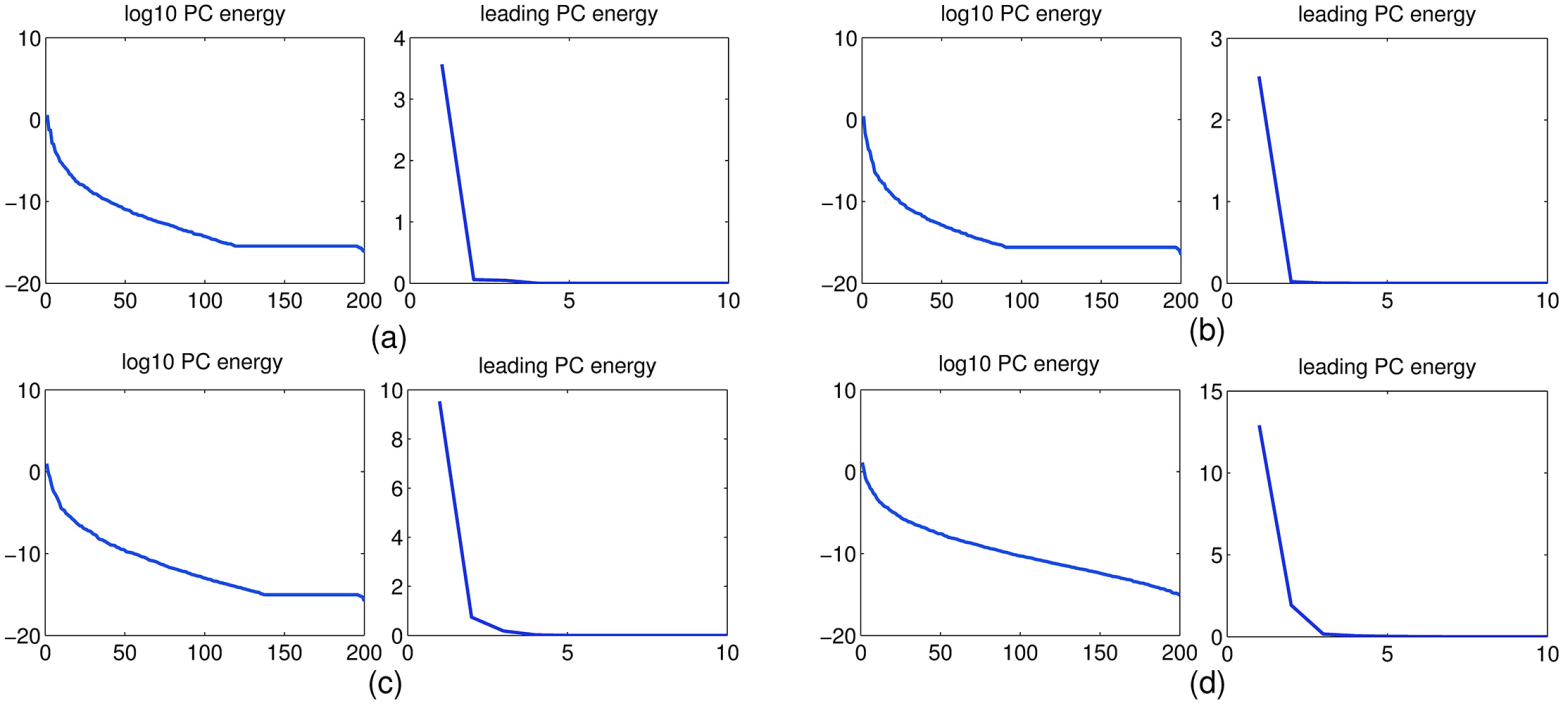
Principal component energy analysis of reservoir states for four different networks. Log PC energy: log10 of reservoir signal energies in the principal component directions. Leading PC energy: The top ten signal energies in linear scale. (a) ESN (b) C-ESN (c) LI-ESN (d) C-LI-ESN.

In addition a principal component analysis (PCA) of the 200 reservoir signals is conducted as shown in Fig 8. Concretely, the reservoir state correlation matrix is estimated by **R** = **XX**^**T**^/*L*, and its SVD **UΣU**^**T**^ = **R** is computed, where the columns of **U** are orthonormal eigenvectors of **R** (the principal component (PC) vectors), the diagonal of **Σ** contains the singular values of **R**, i.e the energies (mean squared amplitudes) of the principal signal components, and *L* is the sampling length during training phase. Fig 8 shows plots of these PC energies and leading PC energies (i.e. close-up on the top ten signal energies in linear scale). The energy spectra are different from each other and the mean squared amplitudes of both PC energy and leading PC energy of the C-LI-ESN are markedly greater than other three networks, which further illustrate the diversity of dynamic characteristic.

**Fig 9:**
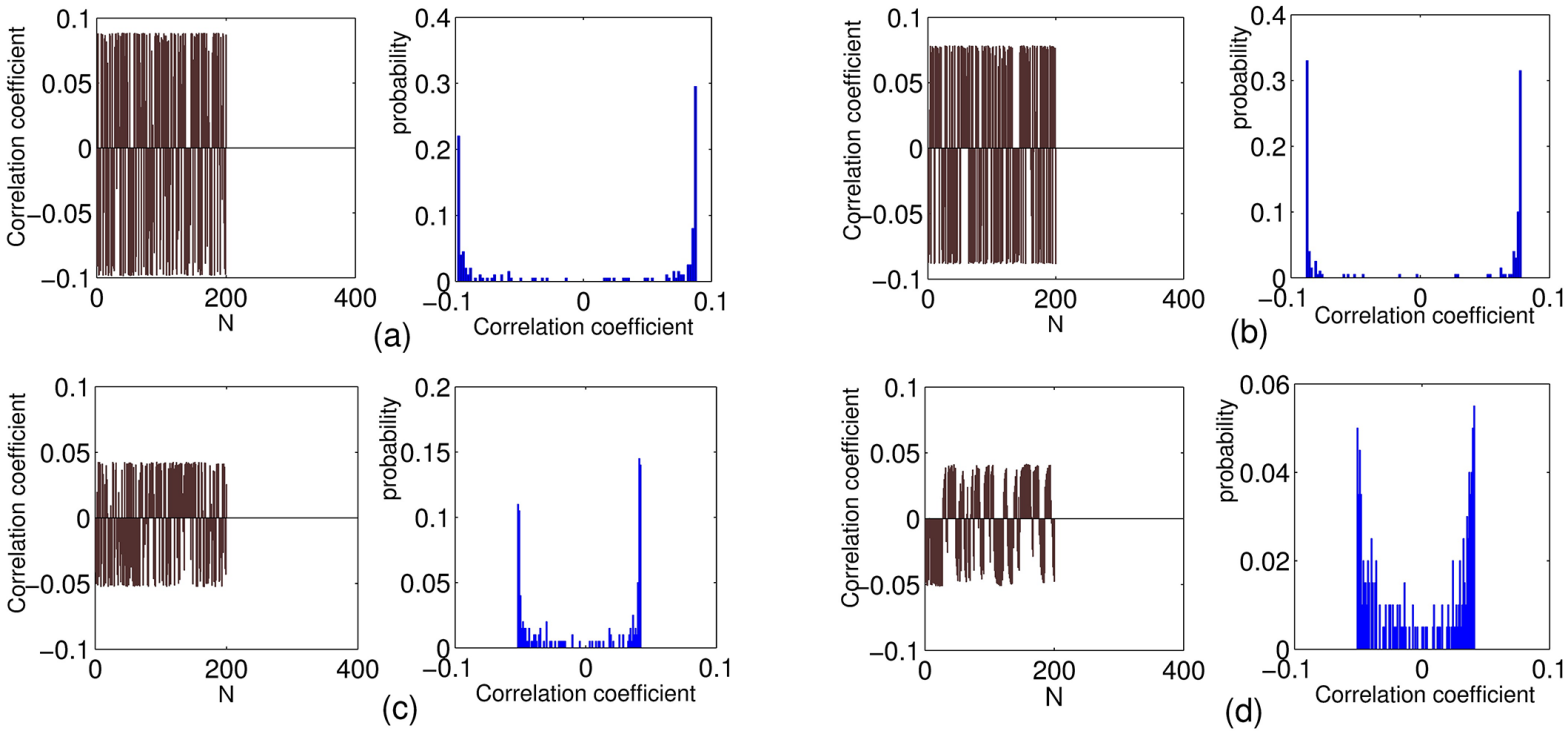
Correlation coefficient and the responding statistical distribution of reservoir states for four networks. (a) ESN (b) C-ESN (c) LI-ESN (d) C-LI-ESN.

Moreover, the correlation of the reservoir units are investigated. The correlation coefficient and the statistical distribution are shown in Fig 9. It shows that the correlation coefficient of C-LI-ESN are much smaller than the others, indicating its low correlation of reservoir dynamic of C-LI-ESN. Therefore, both the rich dynamics and low correlation of reservoir states contribute to the remarkable enhancement of computational performance of C-LI-ESN.

### Capability of nonlinear time series prediction

**Fig 10:**
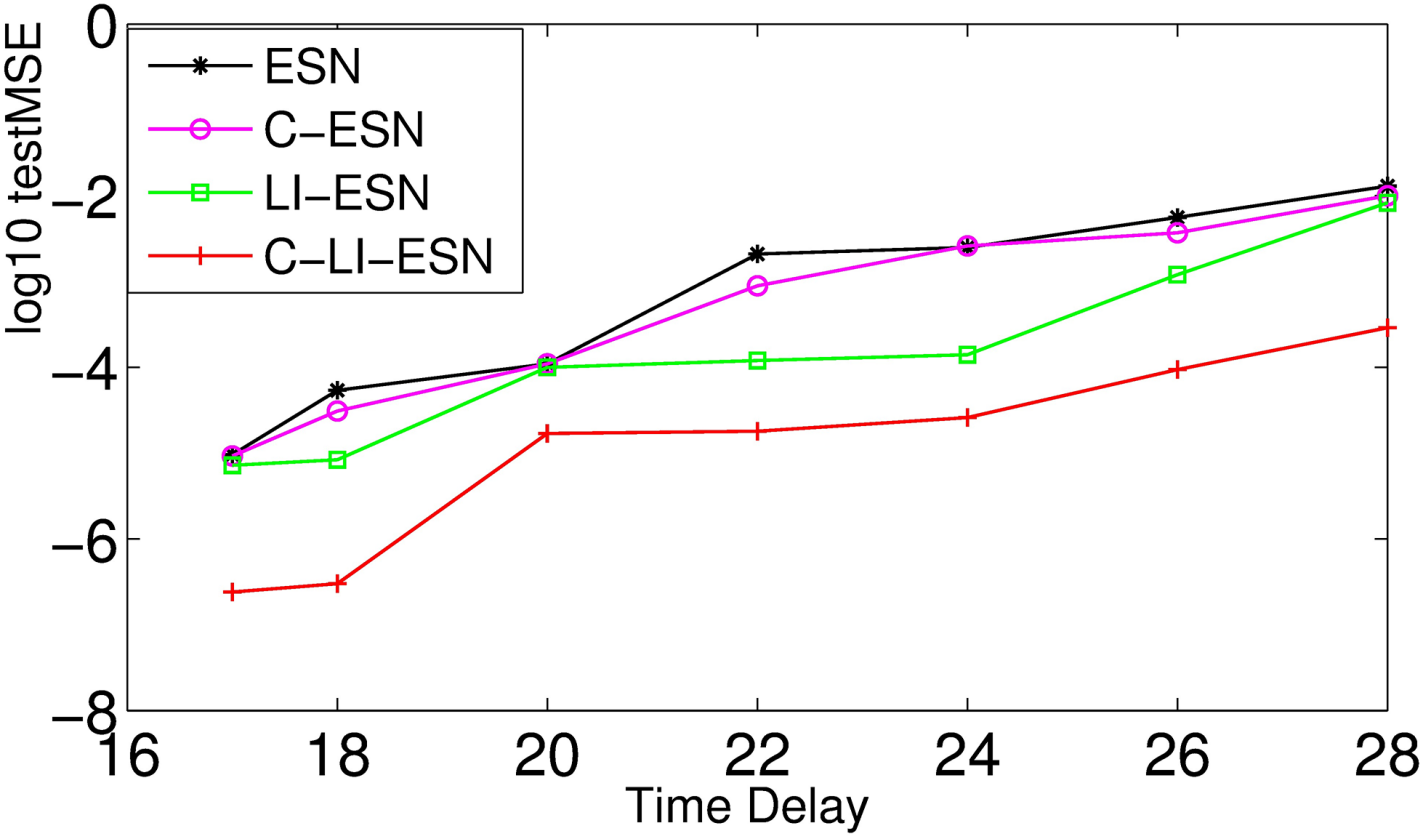
Capability of nonlinear time series prediction. The log10 testMSE of four networks vs the time delay *τ* in the Mackey-Glass system.

It is known that as the time delay *τ* gets larger the nonlinearity of MGS becomes greater as shown in Fig 3. Fig 10 shows that the performance capability of nonlinear time series prediction for all networks are gradually decreasing with time delay varying from 17 to 28 as shown in Fig 10. However, the performance of C-LI-ESN is still obviously superior to the other three networks. The LI-ESN performs better than ESN and C-ESN.

**Fig 11:**
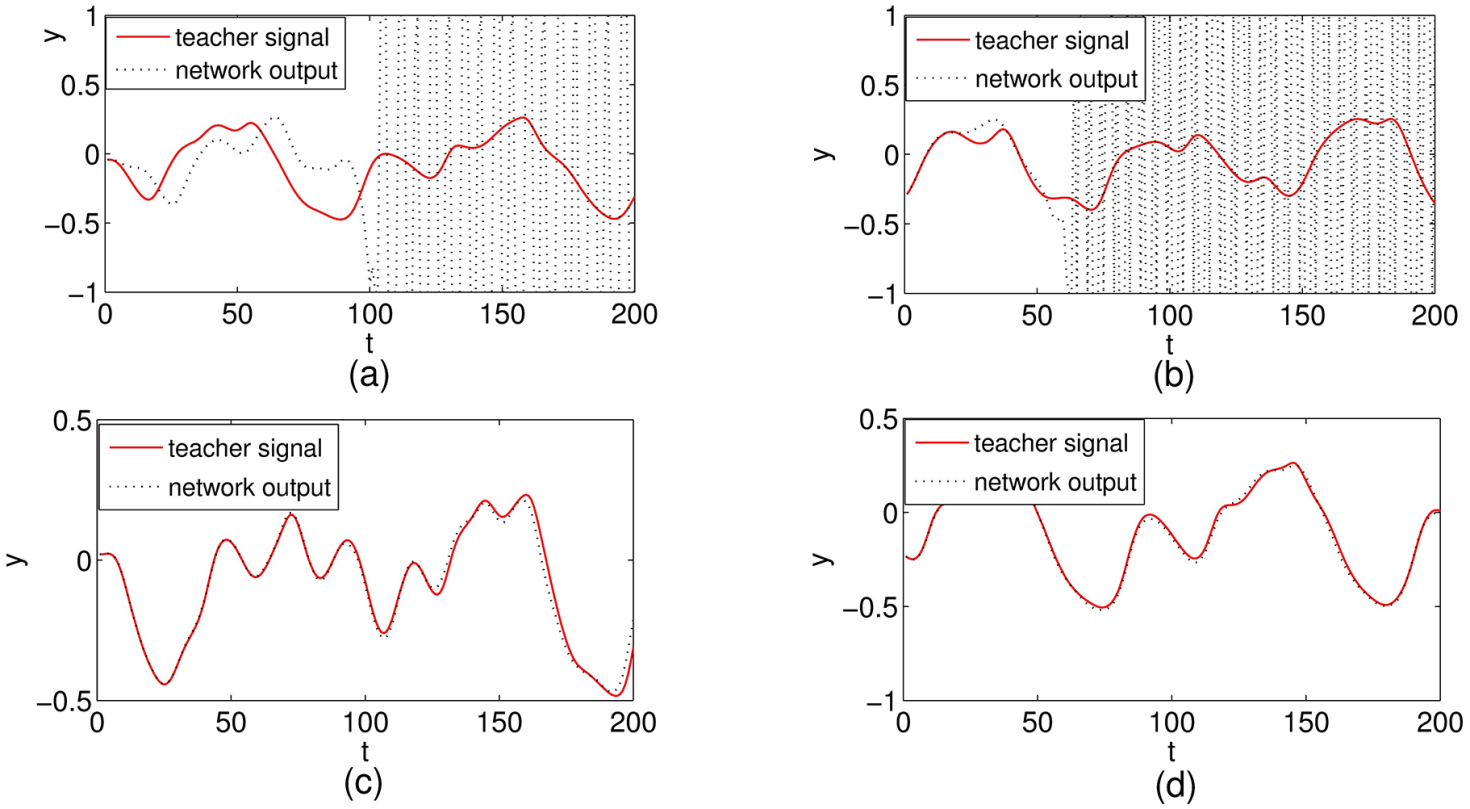
Teacher signal (solid) and network output (dashed) for four networks with the parameter of learning task *τ* = 30. (a) ESN (b) C-ESN (c) LI-ESN (d) C-LI-ESN.

**Fig 12:**
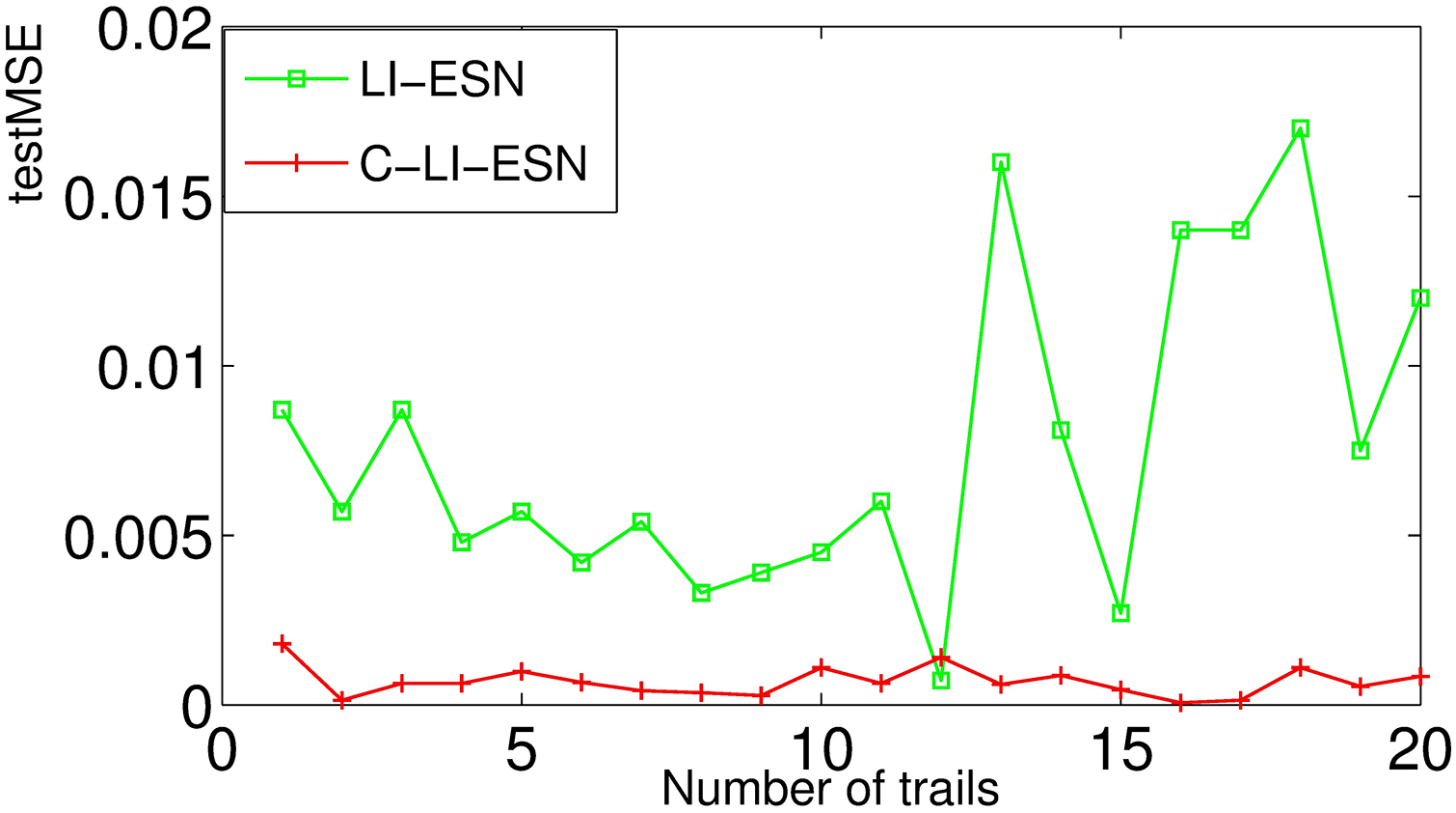
Comparison of *testMSEs* of LI-ESN and C-LI-ESN for the learning task *τ* = 30. The results are obtained by 20 independent realizations.

Specifically, we set the parameter *τ* to be 30 and re-compare the ability of nonlinear time series prediction for different ESN models. In this case, the Mackey-Glass system has significantly high nonlinearity and difficult to be predicted. Our experimental results show that the output of ESN and C-ESN become unstable as given in Fig 11(a) and 11(b). However, the output of LI-ESN and C-LI-ESN can match the teacher signal well as illustrated in Fig 11(c) and 11(d). The performance of these two stable networks is further compared by calculating the *testMSE* as shown in Fig 12. These results demonstrate that when the MGS becomes a high nonlinear system, the abilities of nonlinear time series prediction for all ESNs decline. However, the C-LI-ESN and LI-ESN can show a remarkable advantage than the classical ESN. Again, C-LI-ESN shows the best performance. Both of the memory ability of leaky integrator units and the simple circle topology improve the ability of capturing complex characteristics of the learning tasks.

### The anti-noise ability

**Fig 13:**
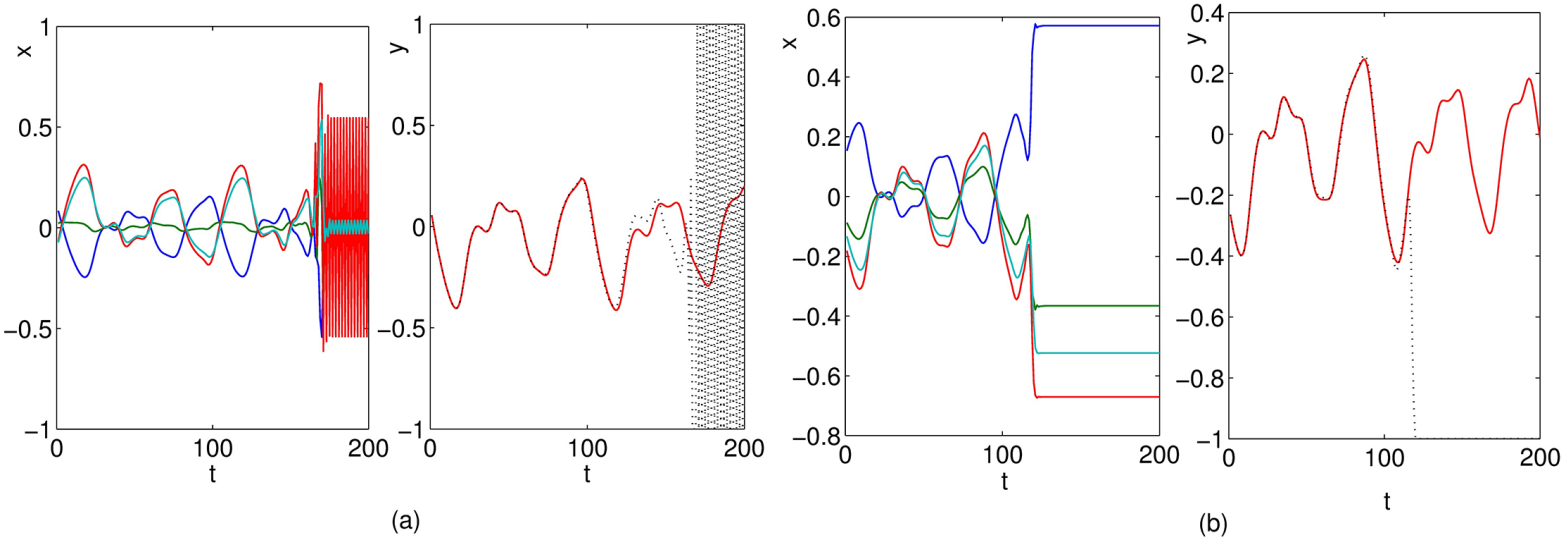
Network output (dashed) diverges from the teaching signal (solid) when internal states become unstable (a) or divergent (b).

In [36], the authors pointed out that the pseudo-inverse training method sometimes bring up non-stationarity phenomenon in some of the independent trials as shown in Fig 13. It can be readily observed that the absolute value of the network output is up to 1 when the reservoir states become unstable or divergent. It is known that injecting noise to the reservoir state during training period can enhance the stability and robustness of trained networks. However, simultaneously the prediction accuracy maybe impaired with the increasing of noise intensity [1].

**Fig 14:**
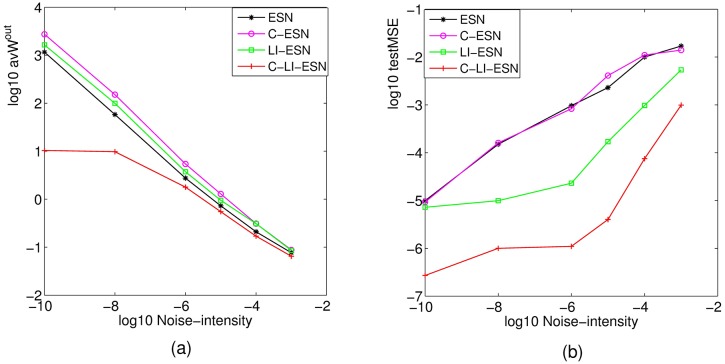
The anti-noise ability for different networks. (a) The average absolute value of output weights vs noise intensity; (b) The testMSE vs noise intensity. All of the data is processed with logarithm.

In this section, the performance of four ESNs with noise intensity varying from 1e-10 to 1e-3 are compared in Fig 14. The mean absolute value of output weights and the testMSE are calculated with the increasing of noise intensity for four ESNs, respectively. In Fig 14(a), since the noise is also an input signal, the increase of input intensity would lead to the decrease of output weights of four networks fitted by the regression training method. Fig 14(a) also shows that the learnt output weights of ESN, C-ESN, LI-ESN are in the order of 100 and even larger when noise intensity is smaller than 1e-7; when the intensity is in the range of 1e-6—1e-3, the output weights of three networks achieve a reasonable range; While for C-LI-ESN, the output weight are always kept in proper range even if the noise intensity is quite weak. From Fig 14(b), we can observe that the C-LI-ESN can performance much better than the other three networks during the whole range of noise intensity. The prediction accuracy of four networks all decline with the increasing of noise intensity due to the increase of task complexity; However, since noise is also an input signal, the increase of input intensity would lead to the decrease of output weights fitted by the regression training method. Actually, adding noise during training is an effective method to reduce output weights, but impair the desired accuracy as well. This result is consistent with the observation in [1], where it also mentioned that the stability of ESNs can be enhanced (i.e. the output weight is reduced) by adding appropriate intensity of noise, but the computational accuracy is depressed.

## Conclusion

In this paper, a new ESN model based on circle topology and leaky integrator units is proposed. The effect of circle structure and leaky integrator neurons on improving the computational performance of ESN are investigated in detail. Mackey-Glass time series is used to test the performance of four ESN networks: classical random ESN with sigmoid neurons, circle ESN with sigmoid neurons, random ESN with leaky integrator neurons, circle ESN with leaky integrator neurons. Comparative simulation experiments including the prediction capability, the capability of nonlinear time series prediction, the anti-noise ability are conducted respectively. The obtained experiment results show that our circle leaky integrator ESN has much better prediction accuracy than other three ESNs due to the rich dynamical diversity and low correlation of reservoir states. Moreover, the proposed C-LI-ESN has much stronger ability to approximate nonlinear dynamics and resist noise than other networks, especially the conventional ESN and ESN with only simple circle structure or leaky integrator neurons. The combination of circle topology and leaky integrator neurons can remarkably improve the performance of echo state network on time series prediction. This work provides an efficient model of ESN with excellent performance and simple network structure, which is very meaningful for the broad application of ESN on various fields. There still remain open problems. For example, strict theoretic analysis for the stability of obvious reservoir models is necessary. Moreover, how to find the optimal values of parameters is also a difficult problem. Further related research and extended application to other real-time or real data tasks would be our future work.
